# Strategy to control magnetic coercivity by elucidating crystallization pathway-dependent microstructural evolution of magnetite mesocrystals

**DOI:** 10.1038/s41467-019-14168-0

**Published:** 2020-01-15

**Authors:** Bum Chul Park, Jiung Cho, Myeong Soo Kim, Min Jun Ko, Lijun Pan, Jin Yeong Na, Young Keun Kim

**Affiliations:** 1 Department of Materials Science and Engineering, Korea University, Seoul, 02481 Korea; 2 Research Institute of Engineering and Technology, Korea University, Seoul, 02481 Korea; 3 Western Seoul Center, Korea Basic Science Institute, Seoul, 03759 Korea; 4 Department of Biomicrosystem Technology, Korea University, Seoul, 02481 Korea

**Keywords:** Magnetic properties and materials

## Abstract

Mesocrystals are assemblies of smaller crystallites and have attracted attention because of their nonclassical crystallization pathway and emerging collective functionalities. Understanding the mesocrystal crystallization mechanism in chemical routes is essential for precise control of size and microstructure, which influence the function of mesocrystals. However, microstructure evolution from the nucleus stage through various crystallization pathways remains unclear. We propose a unified model on the basis of the observation of two crystallization pathways, with different ferric (oxyhydr)oxide polymorphs appearing as intermediates, producing microstructures of magnetite mesocrystal via different mechanisms. An understanding of the crystallization mechanism enables independent chemical control of the mesocrystal diameter and crystallite size, as manifested by a series of magnetic coercivity measurements. We successfully implement an experimental model system that exhibits a universal crystallite size effect on the magnetic coercivity of mesocrystals. These findings provide a general approach to controlling the microstructure through crystallization pathway selection, thus providing a strategy for controlling magnetic coercivity in magnetite systems.

## Abstract (web summary)

Transient metastable intermediates play an important role in the crystallization process. Here, the authors unveil the microstructural changes in magnetite mesocrystals that depend on the intermediate polymorphism and the universal crystallite size effect on the magnetic coercivity of mesocrystals.

## Introduction

Magnetic coercivity behavior is essential for demonstrating magnetism in fine particles and is also a crucial performance indicator for a wide range of electrical and biomedical applications^1–5^. Of many magnetic materials, magnetite (Fe_3_O_4_), which is biocompatible and exhibits superparamagnetic–ferrimagnetic transition depending on its size, has been extensively studied as a diagnostic and therapeutic reagent, leading to advances in the biomedical field^1,6^. To date, shape, size, and composition are used to control magnetic coercivity for applications such as magnetic particle imaging (MPI), which require nonlinear magnetism or magnetic hyperthermia requiring hysteresis loss. However, remarkable advances currently appear unlikely^5^. The microstructure provides a breakthrough in the control of coercivity because crystallite size and inter-crystallite magnetic interaction are closely related to coercivity^1–3^. To verify this at the nanoscale, the magnetic mesocrystal (an assembly of smaller crystallites) is a promising model system for exhibiting collective magnetic coercivity behavior influenced by individual crystallites^7–9^. Magnetic mesocrystals have been reported to exhibit magnetic properties dissimilar from those of single-crystalline particles, depending on the structural arrangement and the magnetic coupling between crystallites. However, we still do not understand the correlation between the microstructure of the mesocrystal and its magnetic properties due to the complexity of the phenomenon. Controlling the crystallite size of mesocrystals at the nanoscale would experimentally reveal this correlation, but chemically controlling the microstructure of mesocrystals synthesized in a bottom-up process still remains a challenge.

The correlation between the microstructure of the Fe_3_O_4_ mesocrystal and its crystallization pathway is an essential underpinning of this study. Understanding the crystallization mechanism in chemical routes is crucial for precise control of the microstructure of Fe_3_O_4_ mesocrystals, which affects magnetic functions. In general, the crystallization mechanism of materials is described by the classical nucleation and growth theory, through which the crystals are spontaneously nucleated in solution and are grown after reaching critical size. For several systems, crystalline phases are not formed directly from solution but by stepwise phase transformations preceded by metastable intermediates^10–12^. Formation of metastable intermediates advances with solubility, and are governed by the Ostwald step rule that the phases with a lower kinetic energy barrier are formed prior to the one with a higher barrier^13^. Recently, several mechanisms besides the classical nucleation and growth model have been observed during the transformation steps of intermediate or crystalline phases^14,15^. In particular, depending on the particle attachment model, mesocrystals comprising assemblies of smaller crystallites frequently exhibit a nonclassical pathway in which crystallization proceeds via attachment of nanometric building blocks (including prenucleation clusters or nanoparticles) and not atom accretion^16,17^.

In these complex processes, the transient metastable intermediate state acts as a precursor to the post-nucleation stage in which the crystalline phase is formed. This intermediate state affects the type and structure of the final crystalline phase, as proposed primarily in calcium carbonate (CaCO_3_), calcium phosphate (Ca_3_(PO_4_)_2_), and calcium sulfate (CaSO_4_) systems^18–20^. The Fe_3_O_4_ phase is also commonly formed stepwise through a series of intermediates down to the lowest-energy rather than being formed directly from ionic precursors^21–23^. For Fe_3_O_4_, various types of ferric (oxyhydr)oxide polymorphs [e.g., α-FeO(OH), β-FeO(OH), and γ-FeO(OH)] and ferrihydrite have been observed as intermediates. Because ferric (oxyhydr)oxide polymorphs represent similar solubilities, the types of intermediates appearing before the Fe_3_O_4_ phase can be significantly varied based on experimental conditions such as pH, additive content, and Fe^3+^ and Fe^2+^ cation concentrations^23–25^. Furthermore, nonclassical crystallization mechanisms, which occur in the formation of ferric (oxyhydr)oxide intermediate or Fe_3_O_4_, can also affect the type of intermediate^26–30^. Baumgartner et al. observed the crystallization process via particle attachment in Fe_3_O_4_ formation and proposed that the energetic stability of primary crystallites can influence the presence of ferrihydrite intermediates^28^. These prior observations provide useful experimental and theoretically indications regarding the role of crystallization pathways. However, we still cannot fully explain the pathways via a universal mechanism, and the evolution of the microstructure during the reaction remains unclear.

In this study, we provide two crystallization pathways of Fe_3_O_4_ mesocrystals in which different ferric (oxyhydr)oxide polymorphs appear separately as intermediates on each pathway. We uncover that the microstructure of Fe_3_O_4_ mesocrystal is affected by which pathway governs the overall reaction and can be chemically controlled by polymorph selection of iron (oxyhydr)oxide. Based on this understanding, diameter and crystallite size of Fe_3_O_4_ mesocrystals are controlled independently. Finally, we report the successful experimental implementation of the crystallite size effect of Fe_3_O_4_ mesocrystal on the magnetic coercivity curves as a function of the diameter. Because Fe_3_O_4_ mesocrystals represent crystallographically ordered aggregate of spheres, we can discuss the collective coercivity behavior, which is difficult to observe in singular particle.

## Results

### Microstructure control via crystallization pathway selection

**Fig. 1:**
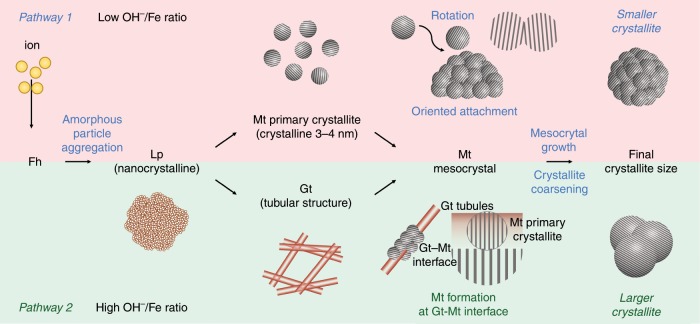
Schematic illustration of the proposed crystallization model of the Fe_3_O_4_ mesocrystal. The two crystallization pathways identified in this study: Pathway 1 (Fh-Lp-Mt: the growth mechanism of Fe_3_O_4_ is oriented attachment), and Pathway 2 (Fh-Lp-Gt-Mt: the growth mechanism of Fe_3_O_4_ is interface-controlled growth). Fh: 5Fe_2_O_3_∙9H_2_O, Lp: γ-FeO(OH), Gt: α-FeO(OH), Mt: Fe_3_O_4_.

We hypothesized that if the Fe_3_O_4_ phase was formed from different ferric (oxyhydr)oxide polymorphs as intermediates, there would be differences in the microstructure of the Fe_3_O_4_ mesocrystal because of the different crystallization mechanisms. If possible, we could chemically control the microstructure by selecting an intermediate among the polymorphs, and eventually, change the crystallite size of the Fe_3_O_4_ mesocrystal. To verify our hypothesis, we observe the crystallization pathways of Fe_3_O_4_ mesocrystals consisting of different crystallite sizes synthesized using the modified polyol method. We identified two crystallization pathways, shown in the schematic illustration (Fig. 1).

lepidocrocite (γ–FeOOH) → magnetite (Fe_3_O_4_) (Pathway 1)

lepidocrocite (γ–FeOOH) → goethite (α–FeOOH) → magnetite (Fe_3_O_4_)(Pathway 2).

In pathway 1, Fe_3_O_4_ primary crystallites with a size of 3–4 nm are produced in the lepidocrocite matrices, and the Fe_3_O_4_ mesocrystals grow such that the crystals adhere to each other in the same crystallographic direction (nonclassical pathway). In pathway 2, Fe_3_O_4_ does not form directly in the lepidocrocite but apparently forms via stepwise transformation from lepidocrocite and goethite, conforming with the Ostwald step rule (classical pathway). Broadly, the entire reaction proceeds concurrently through these classical and nonclassical pathways. However, each pathway produces Fe_3_O_4_ mesocrystal at different stages. In pathway 1, the Fe_3_O_4_ mesocrystal forms at an early stage in the reaction, while in pathway 2, it is nucleated at a later stage in the reaction. Further, the microstructure is affected by the pathway governing the entire reaction and can be chemically controlled. We confirmed that the size of the crystallites formed in pathway 1 is smaller than that of those formed in pathway 2.

### Crystallization processes of sample 1 and sample 2

We compared the formation process and microstructural evolution of Fe_3_O_4_ mesocrystals having the same ~60 nm diameter (*D*) but different crystallite sizes (*C*) of 23 and 43 nm (Sample 1 (S1): *D* = 62 ± 3 nm, *C* = 23 ± 3 nm; Sample 2 (S2): *D* = 61 ± 4 nm, *C* = 43 ± 3 nm) synthesized using Fe:NaOAC:H_2_O ratios of 2:15:150 and 1:3:200, respectively (Supplementary Figs. 1, 2). Uniform 60 nm Fe_3_O_4_ mesocrystals are synthesized in both cases.

**Fig. 2:**
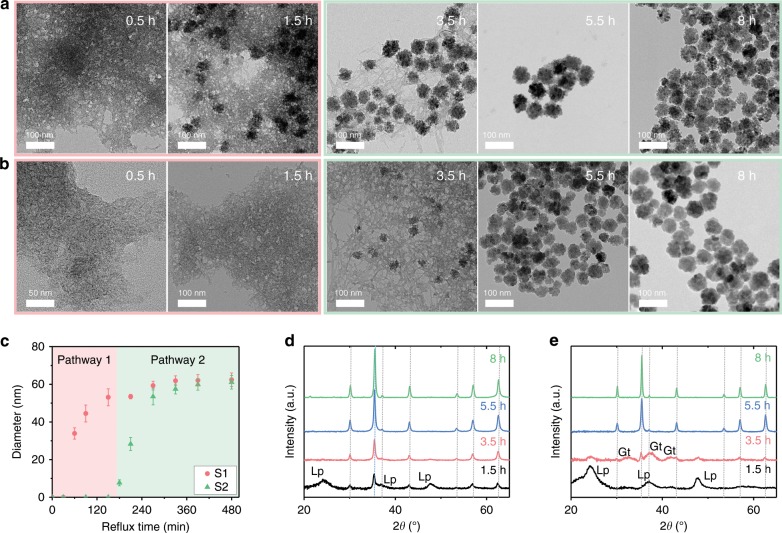
Crystallization of Fe_3_O_4_ mesocrystals in S1 and S2 during refluxing. **a**, **b** TEM images of **a** S1 and **b** S2 after 0.5, 1.5, 3.5, 5.5, and 8 h of refluxing. **c** Growth of Fe_3_O_4_ mesocrystals in S1 and S2. Red (pathway 1) and green (pathway 2) indicate growth from different ferric (oxyhydr)oxide polymorphs. Diameters are mean values of the Gaussian function measured from *n* > 200 mesocrystals in TEM images; error bars indicate standard deviations. **d**, **e** XRD patterns of **d** S1 and **e** S2. Lepidocrocite (Lp), goethite (Gt), and magnetite (Mt) are indexed using ICDD no. 00-044-1415, 00-029-0713, and 01-086-1344 (gray dotted line), respectively.

Figure 2 presents the formation process of S1 and S2 at various reflux times. The Fe_3_O_4_ mesocrystals grow much more rapidly in S1 than in S2 (Supplementary Figs. 3, 4). Fe_3_O_4_ mesocrystals of S1 and S2 are synthesized in different reaction stages from different intermediates. Transmission electron microscopy (TEM) results reveal that the cause of this difference in the initiation time of mesocrystal formation is due to the crystallization pathway rather than the reaction rate (Fig. 2a, b).

In S1, the Fe_3_O_4_ mesocrystal grows rapidly in the nanocrystalline intermediate (lepidocrocite) before refluxing for 3 h, after which it grows in the tubular intermediate (goethite). Conversely, in S2, most of the nanocrystalline intermediates are transformed into tubular intermediates rather than Fe_3_O_4_ mesocrystals in the first 3 h of refluxing, and Fe_3_O_4_ mesocrystals began to grow primarily from the tubular intermediate. Both S1 and S2 show a similar crystallization process after 3 h refluxing. Fe_3_O_4_ mesocrystals in S1 grow primarily from a nanocrystalline intermediate before refluxing for 3 h (pathway 1), while that of S2 grows from a tubular intermediate after refluxing for 3 h (pathway 2). This indicates that S1 and S2 were grown through different crystallization pathways: S1 through pathway 1 and S2 through pathway 2.

This phenomenon is well reflected in Fig. 2c which presents the changes in the diameter of the Fe_3_O_4_ mesocrystal over time for S1 and S2. The growth kinetics of S1 shows two-step growth, while that of S2 shows one-step growth. The growth kinetics of each crystallization pathway is clearly distinguishable around 3 h. In S1, the Fe_3_O_4_ mesocrystals grew to 53 nm via pathway 1 and then grew an additional 9 nm via pathway 2. In S2, Fe_3_O_4_ mesocrystals did not form on pathway 1 but grew after 3 h via pathway 2. The formation of Fe_3_O_4_ mesocrystal via pathway 2 begins at similar times in both S1 and S2, which is illustrated by the green region of Fig. 2c.

Based on the X-ray diffraction (XRD) patterns (Fig. 2d, e), we could define the phases of nanocrystalline and tubular intermediates. In XRD patterns of S1 (Fig. 2d), the Fe_3_O_4_ [International Center for Diffraction Data (ICDD) no. 01-086-1344] and ferric (oxyhydr)oxide phases coexisted after 1.5 h of refluxing. The broad diffraction peaks are assigned primarily to poorly crystalline lepidocrocite (ICDD no. 00-044-1415). With increasing reflux time, the peaks of the Fe_3_O_4_ phase become prominent and sharpened. For S2, we identify the tubular intermediate phase observed primarily in pathway 2 (Fig. 2e). At 3.5 h, the peak from lepidocrocite decreases, and that from goethite (ICDD no. 00-029-0713) appears, indicating that tubular ferric (oxyhydr)oxide intermediates could be assigned to goethite, a polymorph of lepidocrocite.

### Crystallization-pathway-dependent microstructural evolution

**Fig. 3:**
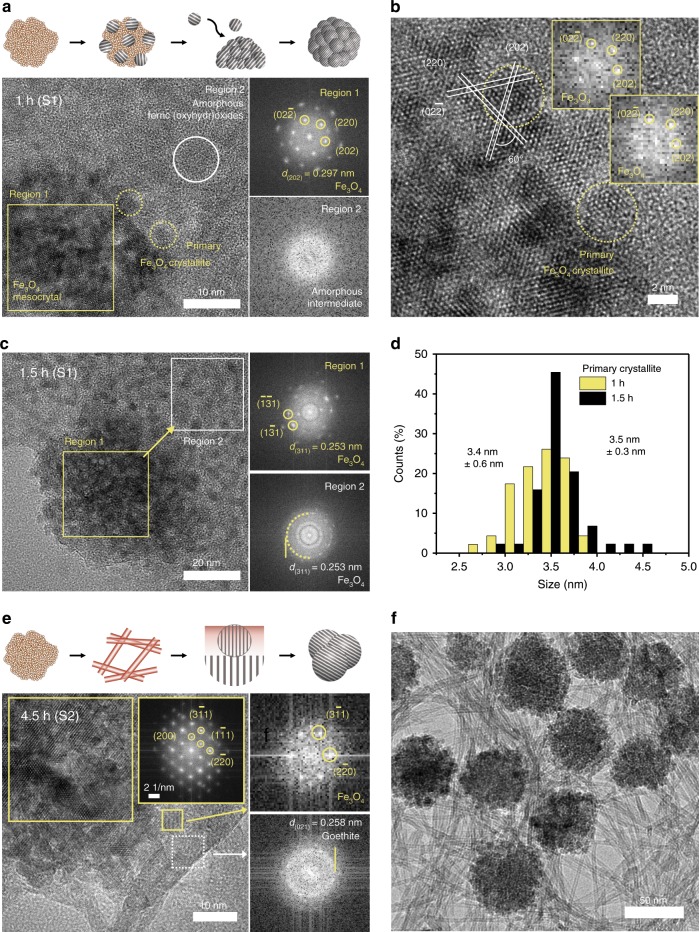
Microstructural analysis of crystallization pathways. **a** TEM analysis of Fe_3_O_4_ mesocrystals of S1 after refluxing for 1 h. **b** HRTEM image showing FFT images of primary crystallites attached to the surface of mesocrystals in S1 after refluxing for 1 h. **c** TEM images and FFT patterns of S1 after refluxing for 1.5 h. The Regions for FFT patterns are indicated in each bright field image of **a**–**c**. The phase and corresponding d-spacing of the FFT patterns are denoted in each panel. **d** Sizes of primary crystallites after 1 and 1.5 h of refluxing. A Gaussian distribution is obtained from the measured sizes of *n* = 50 mesocrystals. **e** HRTEM image and FFT patterns of S2 after refluxing for 4.5 h. The FFT pattern shows d-spacing of spots derived from the magnetite and goethite closest to the center. **f** Bright-field image of S2 after refluxing for 4.5 h.

An analysis of the microstructure along each crystallization pathway is presented in Fig. 3. As shown in Fig. 3a, in the early stage of the reaction in S1, Fe_3_O_4_ mesocrystals, comprising spherical primary crystallites in nanocrystalline lepidocrocite intermediates, appear after 1 h of refluxing. In region 1, the fast Fourier transform (FFT) pattern of the Fe_3_O_4_ mesocrystal appears as a spot pattern, indicating that the primary crystallites have slightly tilted and almost identical orientations. The spots closest to the center are indexed as the (220) family of planes of Fe_3_O_4_. This is the characteristic phenomenon of mesocrystal formation in which the building units are crystallographically aligned^31^. Figure 3b shows that the FFT pattern of the primary crystallite on the Fe_3_O_4_ mesocrystal surface has the same crystallographic orientation as the core. The Fe_3_O_4_ mesocrystal and primary crystallites are attached along the direction of the (220) planes. In region 2, spherical intermediates appear and exhibit an unclear FFT pattern attributed to short-range order (Fig. 3a). The SAED pattern of the intermediates also forms a wide ring pattern, indicating that the intermediate phase exhibits poor crystallinity (Supplementary Fig. 5). After 1.5 h of refluxing, the growth of Fe_3_O_4_ mesocrystals through oriented attachment of the primary crystallites becomes prominent (Fig. 3c). Comparing the samples that reacted for 1 and 1.5 h, the number of primary crystallites around the mesocrystals increases more rapidly as the reaction time increases. The FFT results gradually change from a single to polycrystalline ring pattern indexed as Fe_3_O_4_ with increasing distance from the mesocrystal. This is because the primary crystallites are randomly oriented at the periphery of the mesocrystal, unlike those on the surface of the mesocrystal. Furthermore, we measured the number and size of primary crystallites in the TEM images and observed that the size of the primary crystallites is fixed at 3.5 ± 0.3 nm, but their number increases 2.8 times as the Fe_3_O_4_ mesocrystal grows (Fig. 3d). This suggests that the growth mode can be attributed to the attachment of primary crystallites and not the accretion of ions or atoms.

We investigate crystallization pathway 2 of the Fe_3_O_4_ mesocrystal by analyzing the microstructure of S2 in which the mesocrystal crystallizes primarily from tubular goethite (Fig. 3e). We observed a consistent phenomenon in the later stage of the reaction in S1 (Supplementary Fig. 6). The structures of tubular goethite and the Fe_3_O_4_ mesocrystals are clearly distinguishable using high-resolution transmission electron microscopy (HRTEM) after 4.5 h (Fig. 3e). The FFT pattern of the mesocrystals is a single pattern, with the zone axis in the [011] direction. The Fe_3_O_4_ mesocrystals are grown at places where they have an interface with goethite, and the newly formed crystallites follow the crystallographic orientation of preexisting Fe_3_O_4_ mesocrystals. Phases other than goethite rarely appear in the FFT patterns away from the mesocrystals. The goethite intermediate exhibits a tubular structure with an external diameter of 2.5–4 nm, an inner diameter of 1–1.5 nm, and a wall thickness of 0.8–1.6 nm (Supplementary Fig. 7). Because it is difficult for this ultra-thin tubular structure to have a long-range order, the FFT pattern suggests poor crystallinity, with a ring pattern that is associated with the (021) plane. Ferric (oxyhydr)oxides exhibit characteristic Raman peaks depending on their polymorphs, and the Raman spectrum of S2 reacted for 4.5 h has the characteristic peaks of magnetite and goethite (Supplementary Fig. 7) at 680 and 384 cm^−1^, respectively^32^.

Adjacent primary crystallites constituting the Fe_3_O_4_ mesocrystals in both samples are coarsened and densely packed, with the appropriate orientation (Supplementary Fig. 6). The size of the crystallite is determined by the post-coarsening process due to variations in the reactant concentrations, with S1, dominated by pathway 1, having smaller crystallites than that with S2, which is dominated by pathway 2.

### Growth kinetics of crystallization pathways

**Fig. 4:**
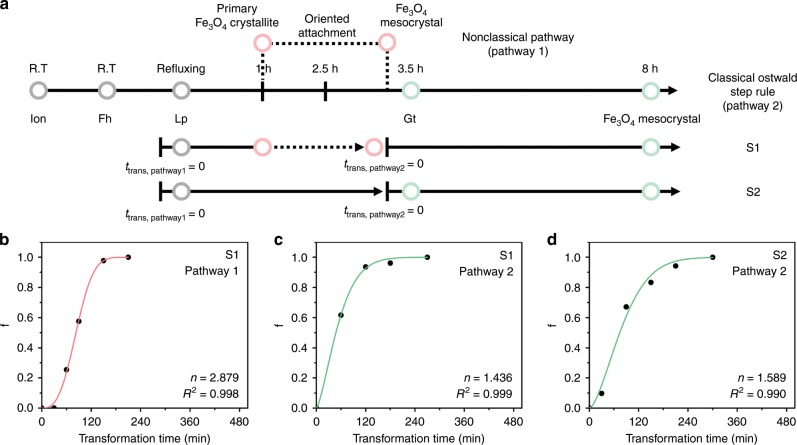
JMAK Kinetics and schematic illustration of the proposed crystallization model of Fe_3_O_4_. **a** Timeline of the entire reaction, and JMAK kinetics. Phases at each time are indicated. Solid and dotted lines represent crystallization pathways 1 and 2, respectively. **b**–**d** Fitting results of JMAK model of Fe_3_O_4_ mesocrystals grown via pathway 1 of S1 (**b**), pathway 2 of S1 (**c**), and pathway 2 of S2 (**d**). Transformation time (*t*_trans_) = 0 is defined as the starting point of each process. Red and green lines indicate the crystallization pathway 1 and 2, respectively.

As discussed in the preceding sections, two coexisting pathways are competitively responsible for the crystallization process of Fe_3_O_4_ mesocrystals. They originate from different mechanisms and participate in different stages of the reaction to form Fe_3_O_4_ mesocrystals that exhibit distinct growth kinetics. Because the nucleation and growth of Fe_3_O_4_ mesocrystals can be explained through phase transformation under isothermal conditions, from ferric (oxyhydr)oxide intermediates to the Fe_3_O_4_ phase, we can gain more insight into the crystallization of Fe_3_O_4_ mesocrystals in S1 and S2 using the Johnson–Mehl–Avrami–Kolmogorov (JMAK) model (Fig. 4)^33^. This model has been extensively applied to phase transformation and polymorphic transformation of solid phase that occurs via nucleation and growth. It is expressed as $f = 1 - \exp [ - \left( {kt} \right)^n]$, where *f* is the volume fraction of the transformed volume, *t* is reflux time, *k* is a constant, and *n* is the Avrami exponent. The Avrami exponent is written as $n = a + \left( {b \times c} \right)$, where *a* is the time-dependent nucleation rate (*a* > 0), *b* is the dimensionality of the grown phase (0 < *b* < 3), and *c* is the growth rate (*c* = 0.5 or 1)^33–35^.

Figure 4a shows the timeline of the reaction and the JMAK model. We applied the model separately for each pathway because the kinetics of the transformation depends on the ferric (oxyhydr)oxide polymorph from which the Fe_3_O_4_ mesocrystals originate. Neither S1 nor S2 contains any phase other than Fe_3_O_4_ after the reaction is complete. Thus, we regard *f* as the volume fraction grown at a particular time with respect to the final volume formed at the end of each pathway (Supplementary Fig. 8). We confirmed that growth in each pathway follows the prediction of the JMAK model: the Avrami exponents of S1 (pathway 1), S1 (pathway 2), and S2 (pathway 2) are 2.89, 1.436, and 1.589, respectively (Fig. 4b–c). The *n* value was derived differently based on the crystallization pathway, where the *n* from lepidocrocite → Fe_3_O_4_ was higher than that obtained from goethite → Fe_3_O_4_. Furthermore, *n* values derived from the transformation of goethite → Fe_3_O_4_ in S1 (pathway 2) and S2 (pathway 2) are similar, which implies that the mechanism of each crystallization pathway is accurately reflected in the classical JMAK model. In S1 (pathway 1), there is spherical growth (*b* = 3) of Fe_3_O_4_ mesocrystals via oriented attachment of primary crystallites. It is hypothesized that a diffusion-controlled process (*c* = 0.5) determines the growth rate when primary crystallites approach each other. Here, *a* is deduced to be 1.3–1.4, indicating the accelerated and autocatalytic nucleation rate^35^. This is in good agreement with the TEM results as the Fe_3_O_4_ primary crystallites formed from lepidocrocite gradually increase over time (Fig. 3a–c). Fe_3_O_4_ primary crystallites are increasingly nucleated in nanocrystalline lepidocrocite and attach to each other to grow mesocrystals. Furthermore, the *n* values of pathway 2 for S1 and S2 are similar. Interface-controlled growth (*c* = 1) was confirmed by direct observation of Fe_3_O_4_ mesocrystal growth at the interface with goethite. Because the Fe_3_O_4_ mesocrystal are formed from one-dimensional tubular goethite intermediate and grow along the orientation of preexisting Fe_3_O_4_ mesocrystals, we can thus deduce one-dimensional growth (*b* = 1) and decreasing nucleation rate in pathway 2 (*a* = 0.4–0.6).

### Chemical control of the crystallization pathways

**Fig. 5:**
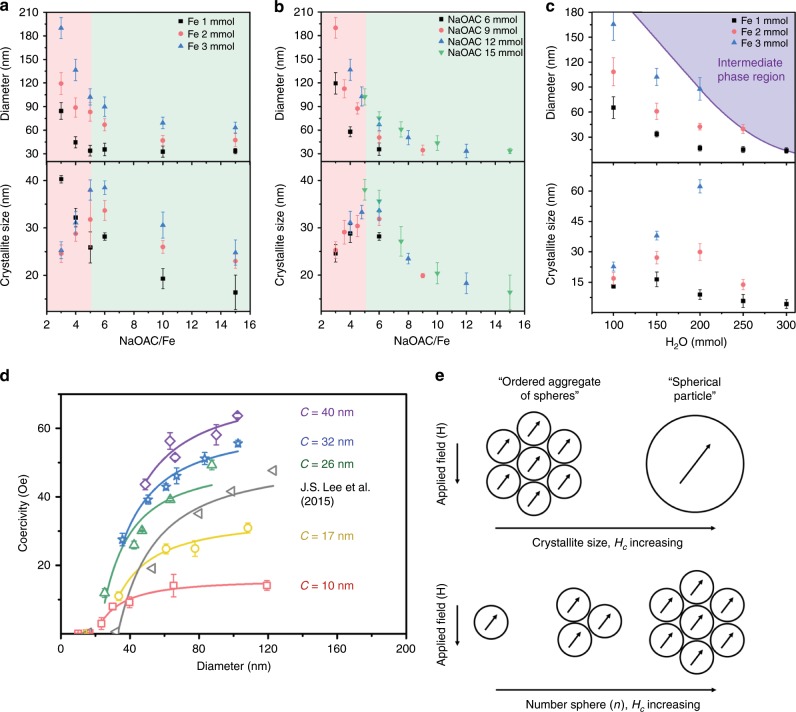
Crystallite size effect on *H*_*c*_ fitting as a function of the diameter of the mesocrystal. **a** Effect of NaOAC, **b** Fe precursor, and **c** H_2_O contents on the diameter and crystallite size of Fe_3_O_4_ mesocrystals. Red and green regions denote the regions where the crystallization pathways 1 and 2, respectively, govern the entire reaction. The solid purple line serves as a guide to the eye. Error bars indicate standard deviations. **d** *H*_c_ fitting depending on the diameter and crystallite size of the Fe_3_O_4_ mesocrystals. We expressed *H*_c_ as a function of the mesocrystal diameter for five groups with different average crystallite sizes. Error bars indicate standard deviations. The smallest mesocrystal for each group is almost a single crystal with a diameter comparable to the crystallite size. The fitted curves are represented by solid lines. **e** Schematic illustration of Fe_3_O_4_ mesocrystal as an ordered aggregate of spheres.

We show that ferric (oxyhydr)oxide polymorphs play an essential role in determining the crystallization pathway of Fe_3_O_4_, which differentiates the microstructures of Fe_3_O_4_ mesocrystals derived via each crystallization mechanism. We propose that the crystallization pathways of Fe_3_O_4_ can be selected by varying the chemical content. Recent experimental and theoretical studies indicate that the solution chemistry affects the free-energy landscape that directly governs polymorph selection^28,36,37^. The free-energy landscape is determined by the ratio of surface to bulk energy, which is a function of solution chemistry. Consequently, even minor variations in reactant type and concentration influence the phase is selected as intermediate among the ferric (oxyhydr)oxide polymorphs, which have different solubilities^23,38,39^. The graphs in Fig. 5a–c represent the effect of the chemical content including FeCl_3_·6H_2_O, NaOAC, and H_2_O on the diameter and crystallite size of the Fe_3_O_4_ mesocrystals. The modified polyol method has been widely used for a decade, but we were able to observe changes in the diameter and crystallite size over a wide range of chemical content and developed a comprehensive understanding of the synthesis process^7,40,41^. Here, we focused on how crystallite size varies with NaOAC/FeCl_3_·6H_2_O ratio and H_2_O content in terms of which crystallization pathway covers the entire reaction (see Supplementary Section 13). Increasing the content ratio of FeCl_3_·6H_2_O (the Fe precursor) to that of NaOAC and H_2_O (the OH^−^ sources) can lead to a reduction in crystallite size, implying that pathway 1 gradually accounts for the entire reaction. The reason could be the effect of excess Fe^3+^ cations, which could be reduced to Fe^2+^ by the subsequent reaction with ethylene glycol. As the Fe^2+^ concentration increases, it is adsorbed onto the surface of the iron (oxyhydr)oxide phases, and it transfers electrons to the bulk iron (oxyhydr)oxide, thus promoting nucleation of Fe_3_O_4_^21,22,42^. As shown in Fig. 5c, when H_2_O is added in excess to the NaOAC/FeCl_3_·6H_2_O ratio, the Fe_3_O_4_ phase is no longer formed, and the reaction is blocked at the intermediate phase. This is because excess H_2_O interferes with dehydration in the stepwise phase transformation of pathway 2^43^.

### A model for the magnetic coercivity behavior

Based on an understanding of the reaction, we can precisely control the diameter and crystallite size of Fe_3_O_4_ mesocrystals (Supplementary Figs. 9–12) and analyze the effect of crystallite size on *H*_c_ (Fig. 5d). Figure 5d presents the *H*_c_ variation of Fe_3_O_4_ mesocrystals as a function of diameter below *D*_s_ for five groups representing different crystallite sizes; the crystallite size of each group of mesocrystals is kept constant. *D*_s_ is the critical size at which a particle changes from single magnetic domain to multiple domains and is 105 nm for Fe_3_O_4_^7^. All the groups fit the empirical equation $H_{\mathrm{c}} = g + \frac{h}{{D^{3/2}}}\left( {{\mathrm{when}}\;D \, < \, D_{\mathrm{s}}} \right)$ well, where *a*, *g*, and *h* are constants, and *D* is the mesocrystal diameter^2^. *H*_c_ shows a consistent relationship with *D*^−3/2^ as it decreases in inverse proportion to the square root of the volume, like in Sharrock’s time-dependent *H*_c_ expression^44^. The empirical constant *g* increases with increasing crystallite size, while *h* decreases (Supplementary Table 1). The *H*_c_ curve of the group with larger crystallites increases more steeply. The fitted *H*_c_ curve from our previous study is included for reference^8^, and it increases more rapidly than the *H*_c_ curve in this study because the crystallite sizes of the samples used in the reference increased with increasing mesocrystal diameter. This implies that the *H*_c_ curve fitting we obtained reflects the crystallite change accurately. Because the crystallites are magnetically coupled, in a manner reminiscent of mesocrystals, the Fe_3_O_4_ mesocrystals behave like a single particle. However, the Fe_3_O_4_ mesocrystals are simultaneously influenced by the individual size effect of the crystallite, which is a subunit of Fe_3_O_4_ mesocrystals^1,3,9^. According to Jacobs and Bean’s chain-of-spheres model, the coercive force required for a magnetically coupled chain-of-spheres is lower than that of an ellipsoid with the axial ratio of the Stoner–Wohlfarth model^3^. The Fe_3_O_4_ mesocrystals are an aggregate of crystallites with the same crystallographic orientation and can be represented as ordered aggregate of spheres (Fig. 5e). The ordered aggregate of spheres needs a lower coercive force to reverse the magnetic moment than that for a spherical particle of the same volume. Furthermore, when an aggregate is composed of smaller spheres, *H*_c_ is reduced because the magnetically coupled interface increases. As the number of constituent spheres increases, *H*_c_ gradually increases. This suggests that the chain-of-spheres model can also be adopted in spherical models that neglect shape anisotropy.

## Discussion

We have discovered a new approach to chemically control the microstructure of Fe_3_O_4_ mesocrystals based on the crystallization pathways, which suggests that the polymorphism of iron (oxyhydr)oxide intermediates can affect the crystallization mechanism. We demonstrate that the growth mechanism of Fe_3_O_4_ mesocrystals from different iron (oxyhydr)oxide polymorphs leads to a variation in the microstructure of the mesocrystal characterized by the JMAK model. The former pathway, following oriented attachment, produces a small crystallite, while the latter, following the classical model by stepwise transformation, produces a large crystallite. These two parallel crystallization pathways operate competitively, and we were able to selectively control the ratio of the pathways that govern the entire reaction depending on the NaOAC/FeCl_3_·6H_2_O ratio and H_2_O content. We hereby propose the *H*_c_ model for Fe_3_O_4_ mesocrystals 100 nm or less in size, which facilitates fundamental magnetism in fine particles. To the best of our knowledge, this is the first report of *H*_c_ variation in Fe_3_O_4_ mesocrystals synthesized using the same method as a function of both diameter and crystallite size. The Fe_3_O_4_ mesocrystals synthesized via chemical routes, with diameters ranging from a few nanometers to a hundred nanometers and exhibiting soft magnetism, are of emerging technological interests in electrical as well as biomedical applications. This study suggests a reliable design rule where coercivity modulation is desirable.

## Methods

### Synthesis of Fe_3_O_4_ mesocrystals

We use a modified polyol method that has been widely employed to synthesize metal/metal oxide mesocrystals with a controlled diameter in a wide range^7,35^. This method is effective for investigating the prenucleation attachment model of Fe_3_O_4_ mesocrystal formation and explaining changes in the geometric and microstructural sizes. The experimental procedure is as follows: we use iron chloride hexahydrate (FeCl_3_·6H_2_O), sodium acetate (NaOAC), and ethylene glycol (which are commonly used in the modified polyol method) as the Fe precursor, hydroxyl ion supplier, and solvent, respectively. We do not use any other surfactant or modifier. In a typical synthesis of Fe_3_O_4_ mesocrystals, FeCl_3_·6H_2_O, NaOAC, and distilled water are completely dissolved in 50 mL of ethylene glycol under vigorous mechanical stirring to form a yellow–brown turbid solution. FeCl_3_·6H_2_O (>97%, Sigma-Aldrich, Korea), NaOAC (>98.5%, Sigma-Aldrich, Korea), and ethylene glycol (>99.5%, Samchun Chemicals, Korea) are used as received. We add distilled water (Millipore Direct-Q UV 3) to the weighed FeCl_3_·6H_2_O powder to obtain the appropriate concentration. Next, the desired volume of FeCl_3_·6H_2_O aqueous solution is injected rapidly into the ethylene glycol solution containing NaOAC. Here, the ratios of FeCl_3_·6H_2_O, NaOAC, and distilled water should be carefully controlled because they significantly influence the diameter and crystallite size of the final Fe_3_O_4_ mesocrystals. The solution is then refluxed for 8 h, during which period it turns reddish-brown and then slowly becomes black. The temperature is set to 200 °C, but the size of the mesocrystals can be controlled by adjusting the duration of the reaction at temperatures between 70 and 90 °C. After cooling to room temperature, the black sediment is washed upwards of five times using ethanol and distilled water to eliminate any organic and inorganic by-products. To monitor the formation process of the mesocrystals, the experiments are stopped after refluxing for the desired time, and the samples stored at room temperature. For S1, we synthesize the Fe_3_O_4_ mesocrystals following the preceding procedure and using 2 mmol of FeCl_3_·6H_2_O, 15 mmol of NaOAC, 150 mmol of H_2_O, and 50 mL of ethylene glycol. Refluxing is performed at 200 °C, and we stop the experiment at 30 min or 1 h intervals. The reaction mixture is cooled to room temperature and stored at 4 °C in a refrigerator. S2 is prepared in the same way as S1, but different amounts of the precursors are used, as follows: 1 mmol of FeCl_3_·6H_2_O, 3 mmol of NaOAC, 200 mmol of H_2_O, and 50 mL of ethylene glycol. We adjust the concentration of each precursor individually to analyze the changes in the diameter and crystallite size of the Fe_3_O_4_ mesocrystals according to the OH^−^/Fe^3+^ ratio. To analyze the effects of NaOAC, 2, 3, 4, 5, 6, 10, and 15 mmol of NaOAC/FeCl_3_·6H_2_O is used, and the amount of FeCl_3_·6H_2_O is constant at 1, 2, and 3 mmol. To analyze the effects of Fe^3+^, 1, 1.5, 2, 2.5, and 3 mmol of FeCl_3_·6H_2_O is used, with 6, 9, 12, or 15 mmol of NaOAC. Furthermore, the amount of distilled water is varied from 100 to 300 mmol while the NaOAC/FeCl_3_·6H_2_O ratio is maintained at 15, 7.5, or 5. We successfully determined the trends in size change of the mesocrystals and crystallites.

### Characterization of Fe_3_O_4_ mesocrystals

The morphology and microstructural evolution of the crystallization pathways of the Fe_3_O_4_ mesocrystals are determined using analytical TEM (FEI, Talos F200X) at 200 kV. To observe the formation process of Fe_3_O_4_ mesocrystals in S1 and S2 at different times while preserving the samples, a crude solution is used for sampling on a TEM grid without washing or dilution. We drop 6 μL of the solution onto a carbon-coated copper grid. Next, the intermediate and Fe_3_O_4_ mesocrystals are adsorbed on the grid for approximately 3 h in a vacuum desiccator, and the remaining solution is removed via filter paper. The samples for the TEM experiments are prepared by diluting washed samples using absolute ethanol and then placing a drop of the sample solution on a carbon-coated copper grid. The sizes of *n* > 500 Fe_3_O_4_ mesocrystals and *n* > 50 primary Fe_3_O_4_ crystallites are measured from the TEM images. The mean value is obtained by fitting the counts using Gaussian distributions. The crystal structure and crystallite size of the emerging phases are determined using powder XRD (PANalytical, X’Pert ProMPD) with a Cu Kα radiation source (*λ* = 1.5406 Å) at the Korea Basic Science Institute, Seoul Western Center. Measurements are performed in *θ*–2*θ* geometry from 20 to 80° using a 45 kV and 40 mA tube. The average crystallite size is calculated using the Williamson-Hall plot, considering the deformation factors of the samples. The crystallite sizes are determined using the Williamson-Hall plot after the profiles of the samples are fitted with a pseudo-Voigt function, and instrumental broadening is corrected. The XRD peak broadening induced by microstrain $\left( {\varepsilon \approx \frac{{\beta {\mathrm{s}}}}{{\tan \theta }}} \right)$ is considered in estimating the crystallite size. The size and strain broadening at different *θ* positions are analyzed using the Williamson-Hall plot and the uniform deformation model, assuming that the microstrain is uniform in all crystallographic directions and the crystallite size and strain contribute independently to line broadening. The strain and crystallite size are calculated from the slope and *y*-intercept of a linear fitting. Samples S1 and S2 have crystallite sizes of 23 and 43 nm, respectively (Supplementary Fig. 2). Raman spectra of Fe_3_O_4_ mesocrystal and ferric (oxyhydr)oxide intermediates were obtained with a confocal–Raman spectrometer (Renishaw, InVia Raman microscope) equipped with a equipped with a Nd-YAG laser operating at 532 nm and a 1200 lines/mm grating. Vacuum dried samples were mounted onto a glass. The samples were measured at 1 s detector exposure time with 100 spectra accumulations using a low laser power of 1% to avoid any damages in the sample. The magnetic properties of the powdered samples are analyzed by measuring the magnetization curves *M*(*H*) using a vibrating sample magnetometer (Microsense, EV9). We measure *M*(*H*) at 300 K using an applied field of up to 20 kOe.

### JMAK model of crystallization of Fe_3_O_4_ mesocrystals

We tried to demonstrate two observed crystallization pathways using JMAK kinetics. Because neither S1 nor S2 contains any phase other than Fe_3_O_4_ after the reaction is complete, we regard *f* as the volume fraction of Fe_3_O_4_ mesocrystals transformed from the appropriate intermediate. Thus, the JMAK equation yields the following: $f = 1 - {\mathrm{exp}}[ - \left( {kt} \right)^n]$, $\frac{{V_{\mathrm{t}}}}{{V_{\mathrm{f}}}} = 1 - {\mathrm{exp}}[ - \left( {kt} \right)^n]$, where *f* is the volume fraction of the transformed material, *t* is the transformation time, *k* is a constant, and *n* is the Avrami exponent. *V*_t_ is the volume of Fe_3_O_4_ mesocrystals grown at each transformation time, and *V*_f_ is the final volume of Fe_3_O_4_ mesocrystals from each intermediate. The Avrami exponent can be written as follows: $n = a + \left( {b \times c} \right)$, where *a* is the time-dependent nucleation rate (*a* > 0), *b* is the dimensionality of the grown phase (0 < *b* < 3), and *c* is the growth rate (*c* = 0.5 or 1). The value of *a* indicates the nucleation rate as follows: *a* = 0 (no nucleation or preexisting nuclei), 0 < *a* < 1 (decreasing nucleation), *a* = 1 (constant nucleation), *a* > 1 (increasing nucleation). The value of *c* indicates the relationship between time and size based on the growth mode, where *c* = 0.5 for diffusion-controlled growth and *c* = 1 for interface-controlled growth. We apply the JMAK model to each pathway separately. This is possible because the Fe_3_O_4_ growth from lepidocrocite can be distinguished from that from goethite based on time. When Fe_3_O_4_ crystals are derived from lepidocrocite or goethite, the growth kinetics operate differently. For S1, the formation process can be divided into two pathways: an early-stage process (up to 3.5 h), and a late-stage process (from 3.5 to 8 h). As shown in the JMAK model timeline of S1, the Fe_3_O_4_ core grows via pathway 1, and then the Fe_3_O_4_ shell grows via pathway 2 (Supplementary Fig. 8).

For pathway 1 of S1 (from Lp to Fe_3_O_4_, *t*_reflux_ = 0–2.5 h, *t*_trans, pathway1_ = 0–2.5 h):

1 $$\frac{{\frac{4}{3}\pi r_{t_{{\mathrm{trans,pathway1}}^3}}}}{{\frac{4}{3}\pi r_1^3}} = 1 - {\mathrm{exp}}\left[ { - \left( {kt_{{\mathrm{trans}},\;{\mathrm{pathway1}}}} \right)^n} \right]$$

For pathway 2 of S1 (from Gt to Fe_3_O_4_, *t*_reflux_ = 3.5–8 h, *t*_trans, pathway2_ = 0–4.5 h):

2 $$\frac{{\frac{4}{3}\pi r_{t_{{\mathrm{trans}},\;{\mathrm{pathway2}}^3}} - \frac{4}{3}\pi r_1^3}}{{\frac{4}{3}\pi r_2^3 - \frac{4}{3}\pi r_1^3}} = 1 - {\mathrm{exp}}\left[ { - \left( {kt_{{\mathrm{trans}},\;{\mathrm{pathway2}}}} \right)^n} \right]$$

For S2, because the Fe_3_O_4_ mesocrystals are formed primarily from goethite, we considered the refluxing time of 3 h at which Fe_3_O_4_ crystals start to form, as *t*_trans_ = 0. For pathway 2 of S2 (from Gt to Fe_3_O_4_, *t*_reflux_ = 3–8 h, *t*_trans, pathway2_ = 0–5 h):

3 $$\frac{{\frac{4}{3}\pi r_{t_{{\mathrm{trans}},\;{\mathrm{pathway2}}^3}} - \frac{4}{3}\pi r_1^3}}{{\frac{4}{3}\pi r_2^3 - \frac{4}{3}\pi r_1^3}} = 1 - {\mathrm{exp}}\left[ { - \left( {kt_{{\mathrm{trans}},\;{\mathrm{pathway2}}}} \right)^n} \right]$$

## Supplementary information

Supplementary Information

## Data Availability

The data that support the findings of this study are available from the corresponding author upon reasonable request.
