# Supplementary material for: Strategy to control magnetic coercivity by elucidating crystallization pathway-dependent microstructural evolution of magnetite mesocrystals
Source: Nat Commun. 2020 Jan 15;11:298. doi: 10.1038/s41467-019-14168-0 (PMC6962372; doi:10.1038/s41467-019-14168-0)
Supplement: Supplementary file 1 — Supplementary Information [file 41467_2019_14168_MOESM1_ESM.pdf]

Supplementary Information

Strategy to control magnetic coercivity by elucidating crystallization pathway-dependent microstructural evolution of magnetite mesocrystals

Park et al.

SI 1. Specifications of the samples for comparing crystallization pathways

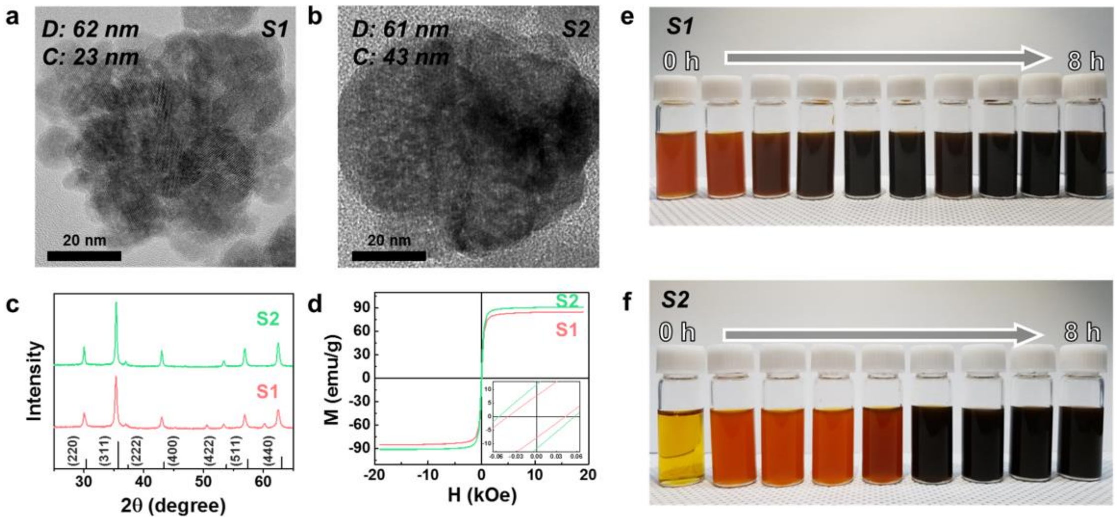

Supplementary Figure 1. Characterization of samples used for monitoring the formation of Fe_3O_4 mesocrystals. **a,b,** TEM images of (a) S1 and (b) S2. The diameter (*D*) and crystallite size (*C*) are marked on the images. **c,** XRD patterns. Magnetite can be indexed using ICDD no. 01-086-1344 (vertical line). **d,** Hysteresis curves of S1 and S2 (inset: high-magnification view of the region near H = 0 Oe). **e,f,** Photographs of solutions at different reflux times (0–8 h); (e) S1 and (f) S2. We prepared 2 mL of each solution immediately after the experiment without a washing procedure.

As shown in the TEM images in Supplementary Figs. 1a and b, the Fe_3O_4 mesocrystals in S1 are composed of smaller primary crystallites than those in S2. The XRD patterns represent the magnetite phase of the inverse spinel structure with crystallite sizes of 23 and 43 nm for S1 and S2, respectively. The S2 sample, with relatively large crystallites, exhibits higher M_s and H_c values than those of the S1 sample. Supplementary Figs. 1e and f show photographs of samples refluxed for 0 to 8 h, with the color gradually changing from orange to black. The color of iron oxides is a clue for distinguishing iron oxide phases; here, it indicates that the black magnetite phase is gradually formed. The reacting solution in Supplementary Fig. 1f (S2) turns black more slowly than that in Supplementary Fig. 1e (S1).

SI 2. Crystallite size analysis using the Williamson-Hall plot

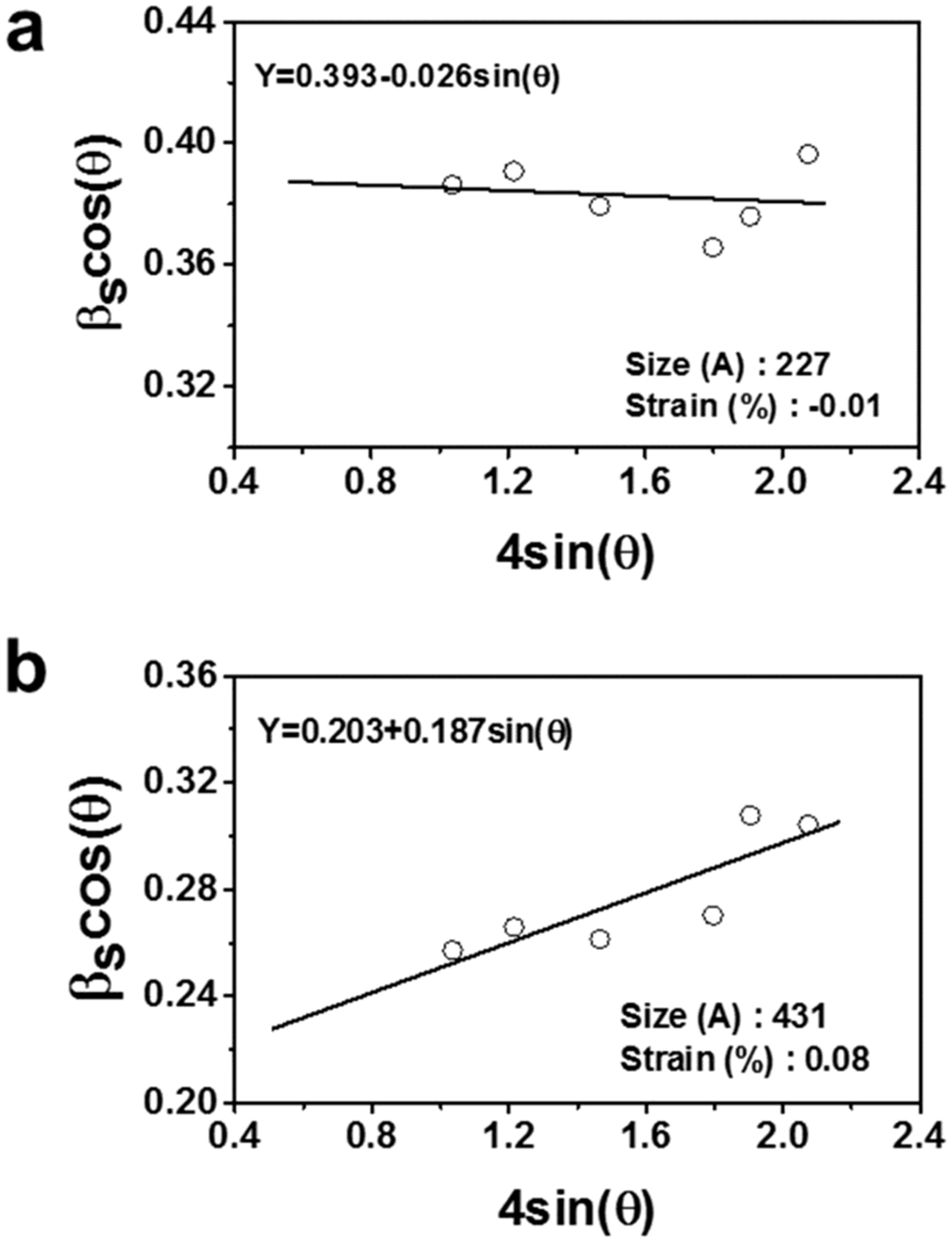

Supplementary Figure 2. Williamson-Hall analysis of the Fe_3O_4 mesocrystals in S1 and S2. From the fitting, the strain is extracted from the slope, and the crystallite size is extracted from the *y*-intercept of the linear fit.

SI 3. Crystallization process of Fe_3O_4 mesocrystals in S1

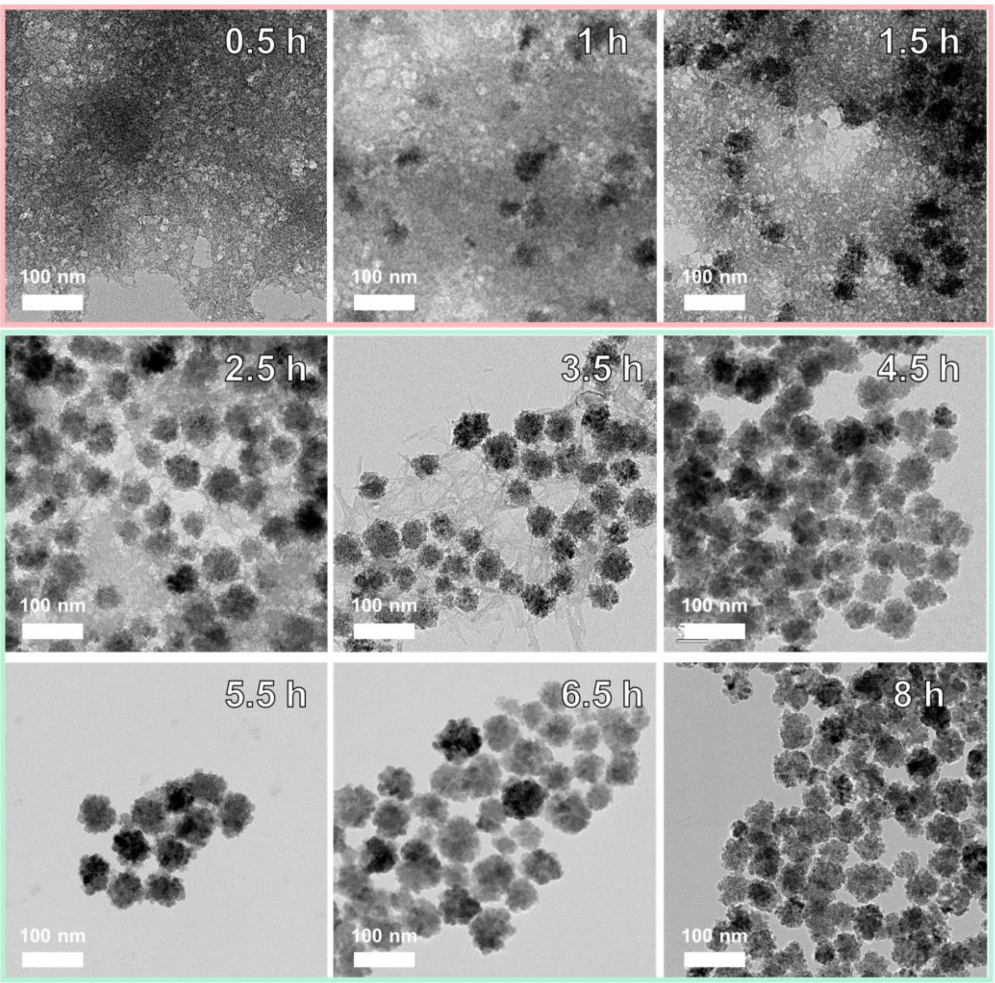

Supplementary Figure 3. TEM images of S1 at different reflux times (0.5–8 h).

Supplementary Fig. 3 shows the formation of Fe_3O_4 mesocrystals over a reflux period of 8 h at 200 °C. The TEM images are distinguishable via two timelines in which Fe_3O_4 phases are formed from different intermediates. The Fe_3O_4 mesocrystals emerge in the nanocrystalline iron (oxyhydr)oxide phase after 1 h and grow gradually up until 2.5 h. After 2.5 h, the nanocrystalline intermediate phase disappears, and the tubular amorphous iron (oxyhydr)oxide phase appears in contact with the Fe_3O_4 mesocrystals. Next, the tubular amorphous iron (oxyhydr)oxide phase disappears, and only Fe_3O_4 mesocrystals are observed until the reflux period ends. No iron oxide phases other than Fe_3O_4 are observed, which confirms that Fe_3O_4 mesocrystals grow as the intermediates are consumed.

SI 4. Crystallization process of Fe_3O_4 mesocrystals in S2

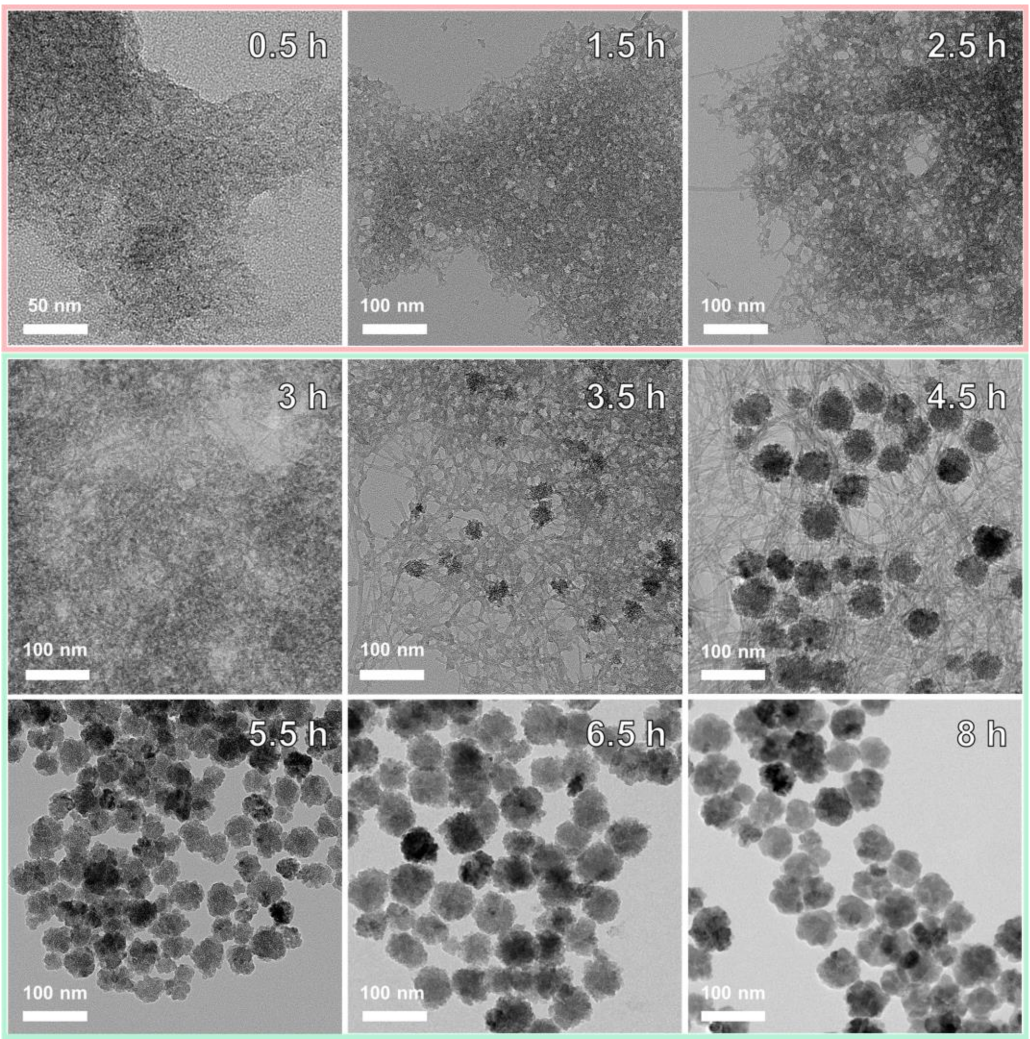

Supplementary Figure 4. TEM images of S2 at different reflux times (0.5–8 h).

As shown in the TEM images in Supplementary Fig. 4, the reaction rate of S2 is much slower than that of S1. The Fe_3O_4 phase appears mainly after 3 h. Fe_3O_4 primary crystallites rarely appear before 3 h of refluxing. For the S1 sample, the primary Fe_3O_4 crystallites are frequently generated directly from nanocrystalline lepidocrocite, while it is difficult to observe the formation of the Fe_3O_4 phase directly from nanocrystalline lepidocrocite in the S2 sample. Most of the growth of Fe_3O_4 mesocrystals in S2 commences after 3 h via Process 2, with the Fe_3O_4 crystals forming by consuming tubular goethite.

SI 5. Microstructural analysis of lepidocrocite intermediate

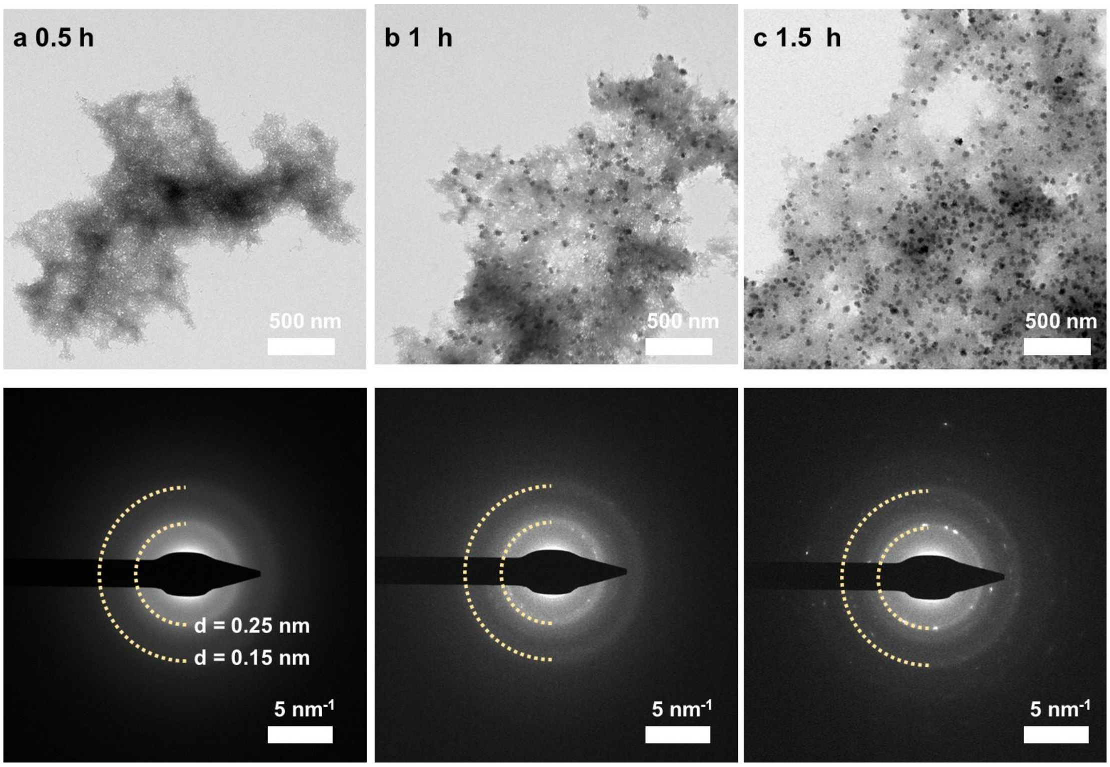

Supplementary Figure 5. Figure R3. Formation of Fe_3O_4 mesocrystal from lepidocrocite. a–c, TEM and SAED pattern of S1 with different refluxing time of 0.5 h (a), 1 h (b) and 1.5 h (c).

SI 6. Microstructural analysis of Pathway 2 and the coarsening process

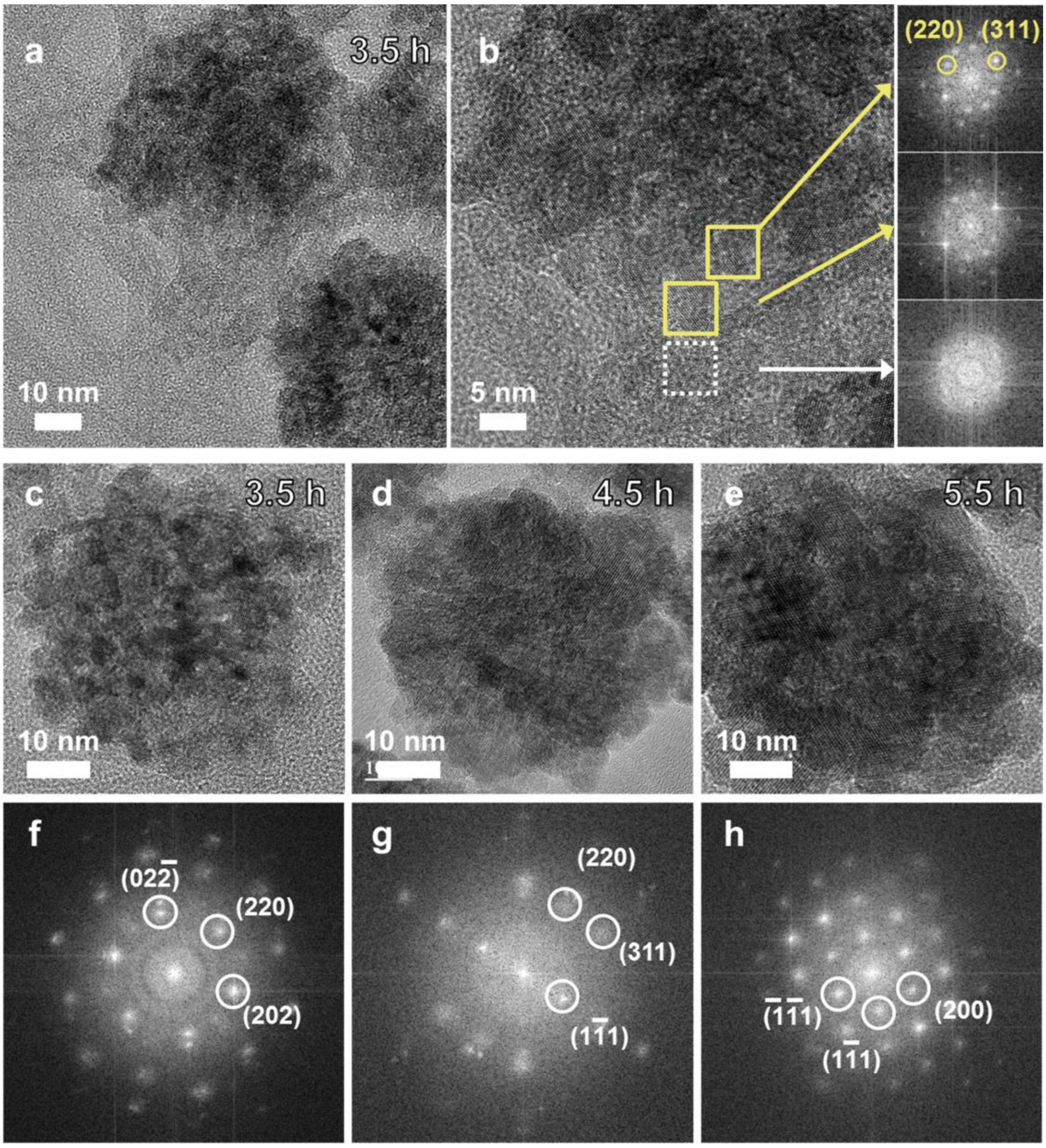

Supplementary Figure 6. TEM analysis of S1 after refluxing for 3.5 h. a,b, Microstructural analysis of Pathway 2 of S1. (a) Bright-field TEM, (b) HRTEM, and FFT images (right) are shown. Rectangles on the HRTEM image indicate regions from which FFT patterns were obtained. **c-h,** Primary crystallite coarsening and crystallite formation (S1). TEM and FFT images of S1 after reflux times of (c,f) 3.5 h, (d,g) 4.5 h, and (e,h) 5.5 h.

Supplementary Figs. 5a and b show the formation of Fe_3O_4 crystals in S1 via Pathway 2. After 2.5 h of refluxing, the nanocrystalline lepidocrocite intermediate gradually disappears, and after 3.5 h, the main intermediate that appears is tubular goethite. This

result indicates that primary Fe_3O_4 crystals are formed in the nanocrystalline iron (oxyhydr)oxide matrix, but another amorphous iron (oxyhydr)oxide phase can form concurrently. As shown by the HRTEM and FFT images in Supplementary Fig. 5b, the Fe_3O_4 crystal emerging from the tubular amorphous iron (oxyhydr)oxide is aligned with the adjacent Fe_3O_4 mesocrystal. However, we do not observe Fe_3O_4 crystals in the tubular region away from the Fe_3O_4 mesocrystal. The Fe_3O_4 crystals grow at the boundary between the tubular intermediate and preexisting Fe_3O_4 mesocrystals.

SI 7. Characteristics of goethite

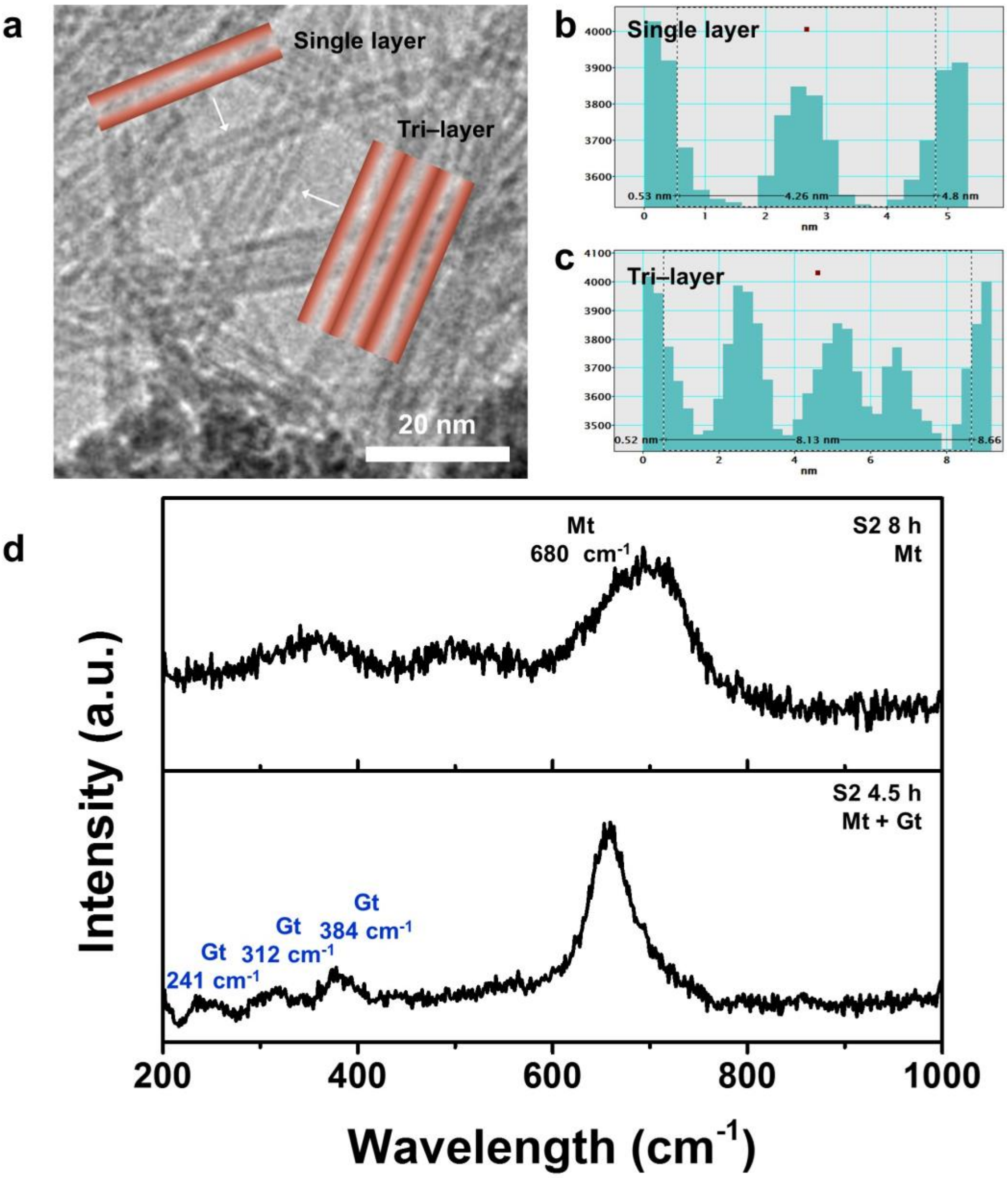

Supplementary Figure 7. Characteristics of tubular goethite intermediate. a, TEM image of tubular goethite intermediate. **b,c,** Line profile of single- and tri-layered goethite intermediate. **d,** Raman spectra of the S2 with different refluxing time of 4.5 and 8h. Goethite (Gt) : α-FeO(OH), Magnetite (Mt): Fe_3O_4.

SI 8. JMAK model and schematic illustration of the entire formation process

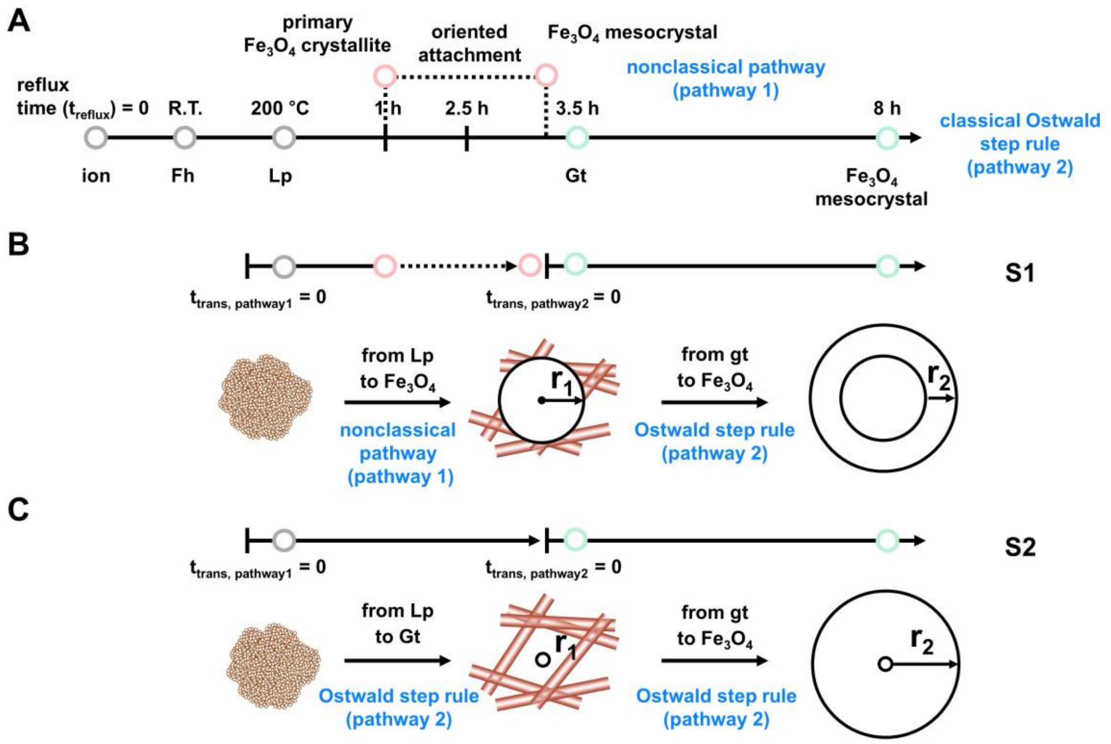

Supplementary Figure 8. Schematic illustration of the JMAK model. a, Timeline of the entire reaction. **b,c,** Timeline of the JMAK model of (b) S1, and (c) S2. We illustrate Fe_3O_4 mesocrystal growth via pathways 1 and 2. We define the transformation time (t_{trans}) as the time required for the growth of Fe_3O_4 mesocrystals via each pathway ($t_{trans,\ pathway1}$ for Pathway 1 and $t_{trans,\ pathway2}$ for Pathway 2). r_1 and r_2 are the final radii of mesocrystals grown from lepidocrocite and goethite, respectively.

The Fe_3O_4 mesocrystals can be formed via different crystallization pathways with distinct mechanisms. They can be formed via stepwise progression from ferrihydrite, lepidocrocite, and goethite (in order of increasing solubility). Crystallization by stepwise phase transformation has been elicited mainly by focusing on the difference in solubility between the intermediates based on the Ostwald step rule. The polymorphs of ferric (oxyhydr)oxide have similar solubility, but the effect of lepidocrocite and goethite on the crystallization pathway of Fe_3O_4 varies. Thus, we observe another pathway on which the Fe_3O_4 mesocrystals emerge earlier than they do via Pathway 2 owing to direct formation of Fe_3O_4 mesocrystals from lepidocrocite, without going through goethite. Both pathways usually operate competitively, but the timing and mechanism of Fe_3O_4 crystal formation

are different for each pathway. On Pathway 1, Fe_3O_4 mesocrystals are formed early in the reaction. They grow by oriented attachment of the primary Fe_3O_4 crystals. When primary crystallites approach each other, the crystallographic orientations of the primary crystallites are rotated, and they share the lattice plane. On Pathway 2, Fe_3O_4 mesocrystals are formed late in the reaction. They grow by the interfacial formation of Fe_3O_4 crystals at the boundary with tubular goethite. The early stage of Pathway 2 involves transformation from nanocrystalline lepidocrocite to tubular goethite. If growth along pathways 1 and 2 occurs, the Fe_3O_4 crystals are grown via Pathway 2 in the later stage, following the crystal facet of preexisting Fe_3O_4 mesocrystal synthesized via Pathway 1.

SI 9. Effect of NaOAC on the formation of Fe_3O_4 mesocrystals

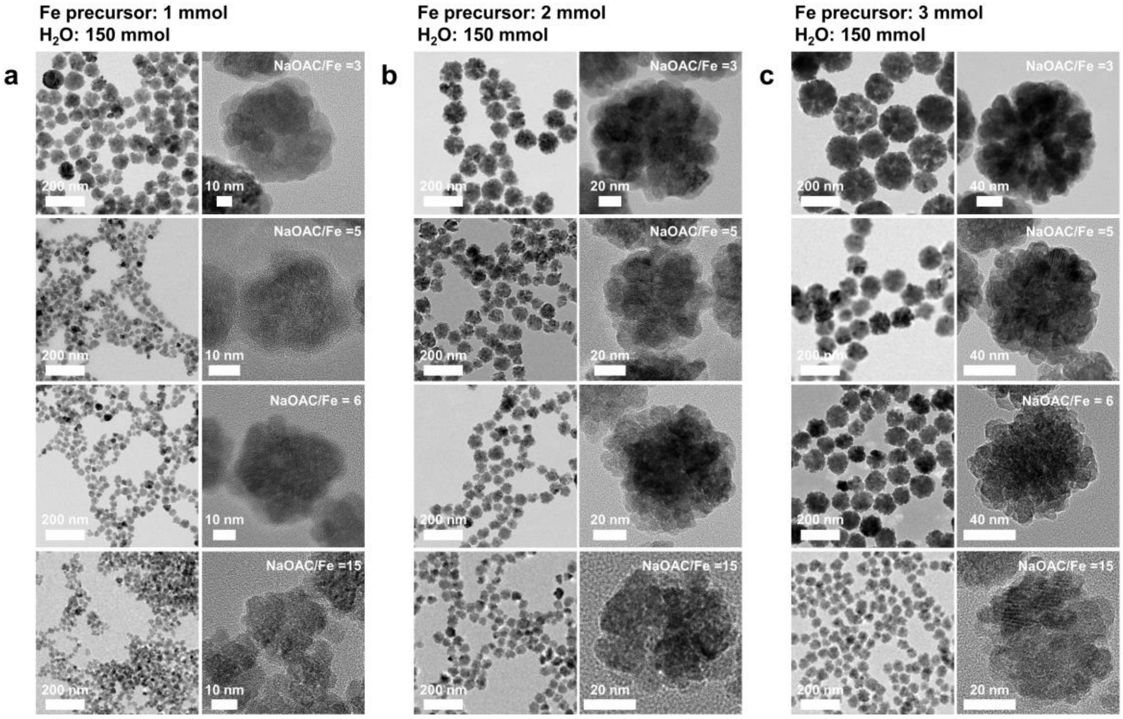

Supplementary Figure 9. TEM images of Fe_3O_4 mesocrystals synthesized with NaOAC/$FeCl_3{\cdot}6H_2O$ at 3, 5, 6, and 15. a–c, The $FeCl_3{\cdot}6H_2O$ content is constant at (a) 1 mmol, (b) 2 mmol, and (c) 3 mmol.

SI 10. Effect of Fe precursor on the formation of Fe_3O_4 mesocrystals

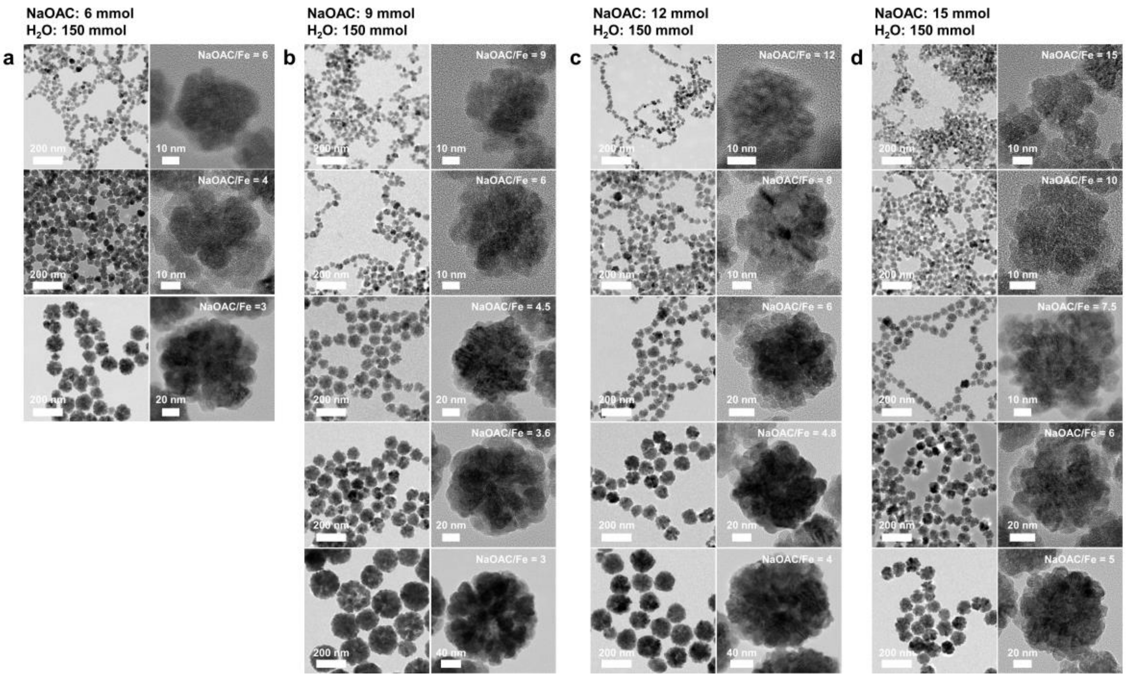

Supplementary Figure 10. TEM images of Fe_3O_4 mesocrystals synthesized at various NaOAC/$FeCl_3$·6H_2O ratios. a–d, The NaOAC content is constant at (a) 6 mmol, (b) 9 mmol, (c) 12 mmol, and (d) 15 mmol.

SI 11. Effect of Fe precursor content on diameter and crystallite size

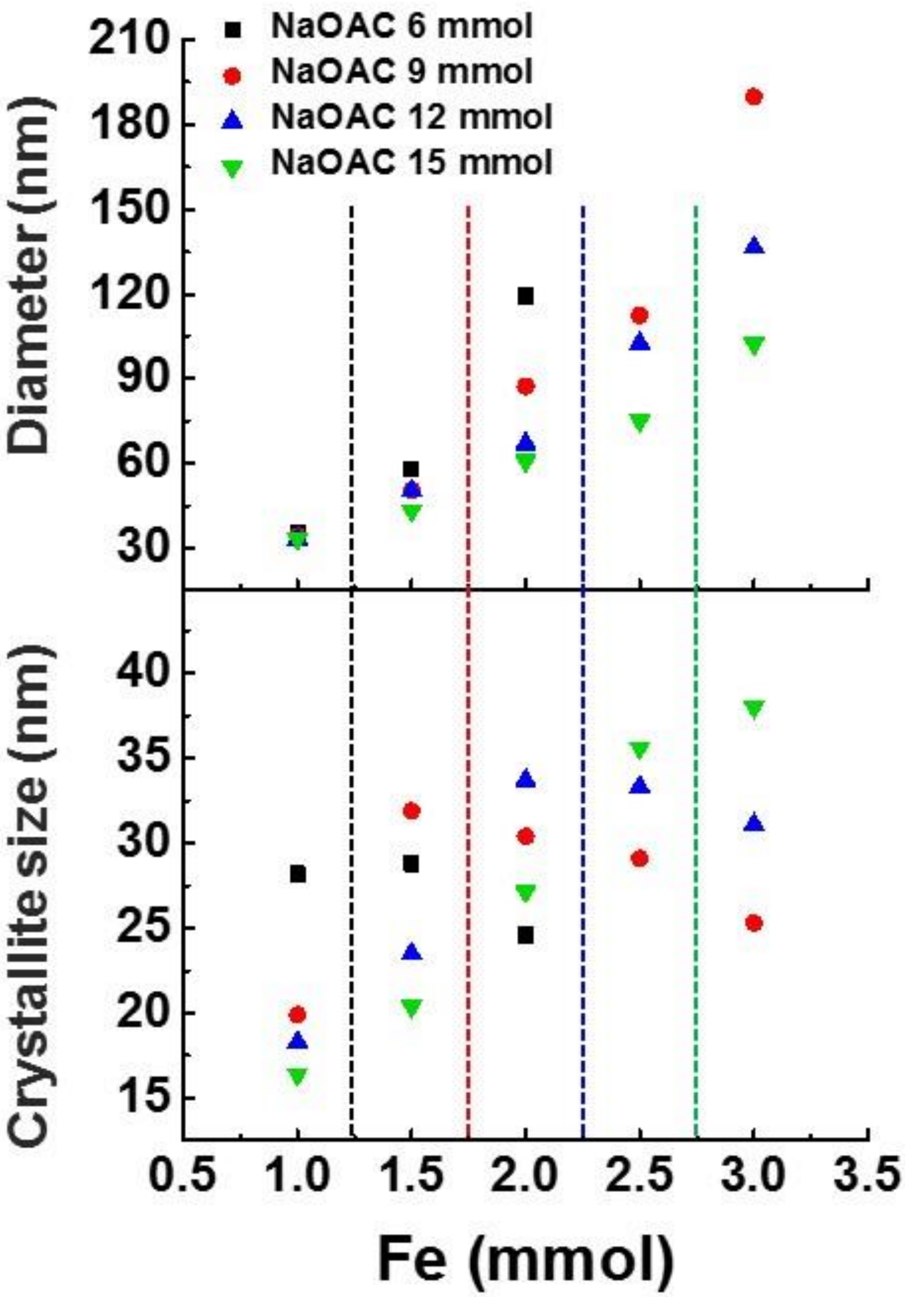

Supplementary Figure 11. Effect of Fe precursor ($FeCl_3 \cdot 6H_2O$) content on the diameter and crystallite size of Fe_3O_4 mesocrystals. The dotted lines denote a NaOAC/$FeCl_3 \cdot 6H_2O$ ratio of 5–6, at which the maximum crystallite size appears, and the diameter begins to increase steeply.

SI 12. Effect of H_2O on the formation of Fe_3O_4 mesocrystals

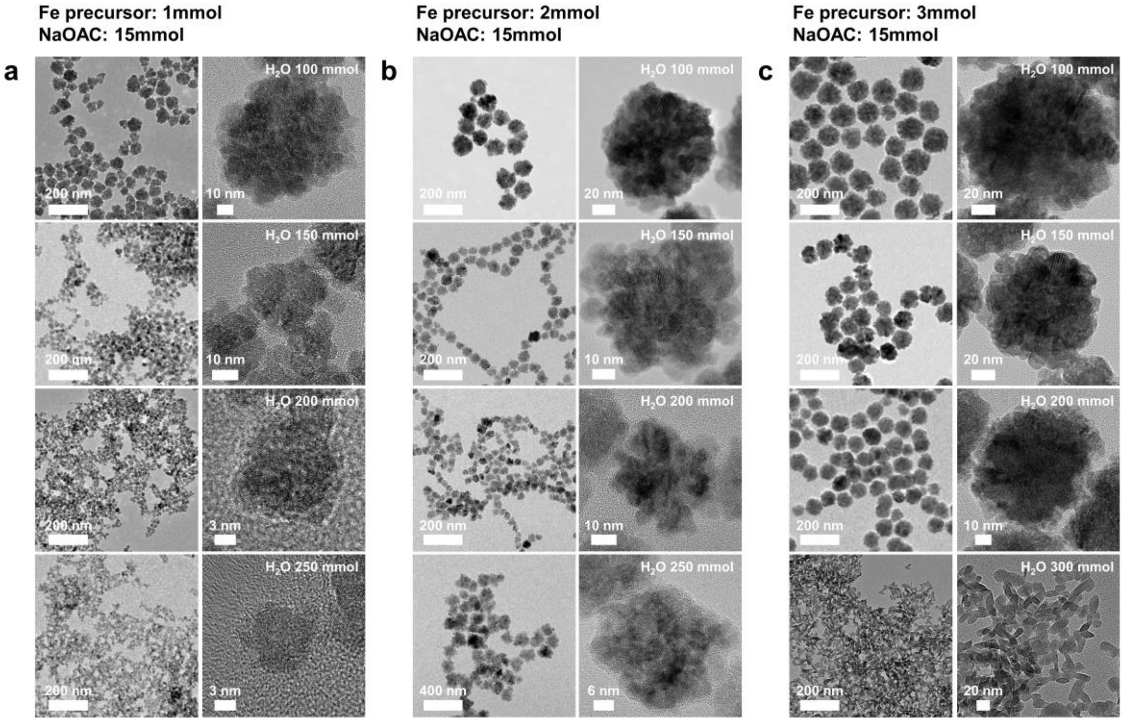

Supplementary Figure 12. TEM images of Fe_3O_4 mesocrystals synthesized with different H_2O contents. a-c, The ratio of NaOAC/$FeCl_3{\cdot}6H_2O$ is constant at (a) 15, (b) 7.5, and (c) 5.

SI 13. Chemical control of crystallization pathway

In Pathway 2 of our experiment, lepidocrocite, goethite, and magnetite were observed in order of increasing solubility, which can be explained using Ostwald's step rule[1]. Because lepidocrocite and goethite have similar solubilities (10^{-7}–10^{-8} M), Pathway 1, via which Fe_3O_4 crystals are formed directly in nanocrystalline lepidocrocite, can occur depending on the chemical composition.

We confirmed variation in the diameter and crystallite size of Fe_3O_4 mesocrystals as the NaOAC concentration increased at constant concentrations of $FeCl_3{\cdot}6H_2O$ and H_2O (150 mmol). Uniform Fe_3O_4 mesocrystals are synthesized with each recipe, as shown in the TEM images in Supplementary Fig. 9. As the NaOAC concentration increases, the diameter tends to gradually decrease. The diameter is inversely proportional to the NaOAC/$FeCl_3{\cdot}6H_2O$ ratio, even if the $FeCl_3{\cdot}6H_2O$ concentration is fixed. The diameter becomes larger when the concentration of $FeCl_3{\cdot}6H_2O$ is increased from 1 to 3 mmol at the same NaOAC/Fe ratio; the graph shifts upward on the *y*-axis. The crystallite size tends to decrease as the NaOAC concentration increases after reaching a maximum value at a NaOAC/$FeCl_3{\cdot}6H_2O$ ratio of 5–6.

We confirmed that the NaOAC/$FeCl_3{\cdot}6H_2O$ ratio can affect the diameter and crystallite size of the Fe_3O_4 mesocrystals even when the Fe precursor content is controlled while the NaOAC and H_2O contents are constant (for TEM images, see Supplementary Fig. 10). To analyze the effects of the Fe precursor, we increased the $FeCl_3{\cdot}6H_2O$ content from 1 to 3 mmol in 0.5 mmol increments while keeping the NaOAC and H_2O contents (150 mmol) constant. As the Fe concentration increases, the diameter of the Fe_3O_4 mesocrystals increases in inverse proportion to the NaOAC/$FeCl_3{\cdot}6H_2O$ ratio. When the NaOAC content increases from 6 to 15 mmol, the diameter decreases more gradually with variation in the NaOAC/$FeCl_3{\cdot}6H_2O$ ratio. Similar to the changes it shows when the NaOAC concentration is varied, the crystallite size exhibits a maximum value at NaOAC/$FeCl_3{\cdot}6H_2O$ = 5–6. When the concentration of the Fe precursor is increased such that the NaOAC/$FeCl_3{\cdot}6H_2O$ ratio becomes smaller than 5–6, the crystallite size tends to decrease, and the diameter tends to increase steeply. These phenomena are presented more clearly in Supplementary Fig. 11, where the *x*-axis represents the Fe content.

To analyze the effect of H_2O, its amount is varied from 100 to 300 mmol while the

$FeCl_3·6H_2O$ and NaOAC contents are kept constant (for TEM images, see Supplementary Fig. 12). The diameter decreases as the amount of H_2O increases. As we add more Fe precursors (from 1 to 3 mmol), the diameter increases. As the amount of H_2O increases, the crystallite size exhibits a maximum value at a certain H_2O content and then tends to decrease. The effect of the H_2O content on the crystallite size is similar to that of the NaOAC content. The reason is that the concentration of OH^- ions is determined by the equilibrium reaction between NaOAC and H_2O. There is a limited range of H_2O content in which Fe_3O_4 mesocrystals can be synthesized. As the $FeCl_3·6H_2O$ content increases from 1 to 3 mmol, the range of H_2O content in which Fe_3O_4 can form becomes narrower. For example, if more than 200 mmol of H_2O is added and the $FeCl_3·6H_2O$ content is kept constant at 3 mmol, goethite (α-FeOOH) and hematite (α-Fe_2O_3) would be synthesized rather than magnetite.

We have shown that two pathways are competitive in the formation of Fe_3O_4 mesocrystals and that the crystallite size differs depending on which pathway is dominant. It can be inferred that the increase in crystallite size when the NaOAC/$FeCl_3·6H_2O$ ratio increases is related to the formation pathway.

The crystallite size decreases when the NaOAC content decreases or the $FeCl_3·6H_2O$ content increases for NaOAC/$FeCl_3·6H_2O$ < 5, indicating that Pathway 1 gradually accounts for the entire reaction. The reason could be the effect of excess Fe^{3+} cations, which could be reduced to Fe^{2+} by the subsequent reaction with ethylene glycol. The addition of Fe^{2+} to preexisting ferrihydrite can determine the subsequent phase[2-4]. Goethite and lepidocrocite appear mainly at low concentrations of Fe^{2+}, and magnetite can be synthesized directly at high concentrations of Fe^{2+}. As the Fe^{2+} concentration increases, it is adsorbed onto the surface of the iron (oxyhydr)oxide phases, and it transfers electrons to the bulk iron (oxyhydr)oxide, thus promoting the growth of magnetite. Here, increasing the supply of NaOAC causes a gradual decrease in the ratio of excess Fe^{3+}, and consequently, the proportion of Pathway 2 on which magnetite is formed by lepidocrocite and goethite gradually increases. Conversely, the supply of the Fe precursor increases the excess Fe^{3+}, and thus Pathway 1 dominates the synthesis of Fe_3O_4 mesocrystals and reduces the crystallite size. The crystallite size tends to decrease for NaOAC/Fe > 5, owing to the effect of the reduced diameter.

It was confirmed that both the crystallite size and diameter tend to behave differently

above and below NaOAC/$FeCl_3 \cdot 6H_2O$ = 5–6. The diameter increases slowly at NaOAC/$FeCl_3 \cdot 6H_2O$ > 5–6 and abruptly at NaOAC/$FeCl_3 \cdot 6H_2O$ < 5–6. Thus, the diameter can also be affected by the formation pathway. In the classical nucleation and growth model, rapid nucleus formation produces larger numbers of small mesocrystals[5]. For Fe_3O_4 mesocrystal synthesis, the concentration of OH^- increases, and hydrolysis and condensation of $FeCl_3 \cdot 6H_2O$ are accelerated, resulting in small Fe_3O_4 mesocrystals.

However, our study suggests that the results depend on the formation pathway. As the NaOAC/$FeCl_3 \cdot 6H_2O$ ratio becomes smaller than 5–6, Pathway 1 dominates, and Fe_3O_4 mesocrystals grow by oriented attachment of primary Fe_3O_4 crystallites. If nucleation of the primary Fe_3O_4 crystallites increases, the chance of contact increases, and the mesocrystals grow larger. For example, as the amount of Fe precursor increases, the amount of excess Fe^{3+} increases, and thus the primary Fe_3O_4 crystallites are nucleated more rapidly, and the mesocrystals quickly grow larger. This differs from the classical pathway in which the diameter decreases as more nuclei form. On the other hand, at NaOAC/$FeCl_3 \cdot 6H_2O$ > 5–6, Pathway 2, on which Fe_3O_4 crystals nucleate and grow at the interface of intermediate and preformed Fe_3O_4 mesocrystals, affects the formation of Fe_3O_4 mesocrystals. Therefore, in this NaOAC/$FeCl_3 \cdot 6H_2O$ range, the size of the Fe_3O_4 mesocrystals can be increased if larger amounts of the intermediate phases are formed by increasing the Fe precursor. Here, this can be explained by a classical model in which mesocrystals grow larger as the number of preformed nuclei decreases.

Furthermore, we found that the water content has an effect similar to that of the NaOAC content, but it has a greater impact on the predominance of Pathway 2. Dehydration occurs during magnetite formation from ferrihydrite to ferric (oxyhydr)oxides. Increases in the H_2O content can interfere with dehydration, resulting in slower reactions (Pathway 2) and larger crystallites.

Supplementary Table S1. Parameters for H_c fitting. The diameter and crystallite size of mesocrystals are determined using TEM images and XRD patterns. H_c values are measured using a VSM at RT.

C = 10 nm			*C* = 17 nm			*C* = 27 nm		
parameters	*g*<br>*h*	16<br>-1419	parameters	*g*<br>*h*	33<br>-4357	parameters	*g*<br>*h*	50<br>-5202
diameter (nm)	crystallite size (nm)	H_c (Oe)	diameter (nm)	crystallite size (nm)	H_c (Oe)	diameter (nm)	crystallite size (nm)	H_c (Oe)
10	4	0.1	15	17	0.05	25	26	11.9
15	6	0.2	34	16	11.0	42	27	25.9
17	9	0.3	61	18	24.8	47	26	30.1
24	15	3.0	78	17	24.9	63	25	39.2
30	15	8.0	108	17	30.9	87	30	49.3
40	14	9.2						
65	13	14.1						
119	17	14.1						

C = 32 nm			*C* = 40 nm			Lee et al. (2015)		
parameters	*g*<br>*h*	60<br>-7229	parameters	*g*<br>*h*	71<br>-9192	parameters	*g*<br>*h*	55<br>-10,000
diameter (nm)	crystallite size (nm)	H_c (Oe)	diameter (nm)	crystallite size (nm)	H_c (Oe)	diameter (nm)	crystallite size (nm)	H_c (Oe)
36	30	27.5	49	40	43.6	16	8	0.25
51	32	39.2	63	38	56.3	32	9	0.57
61	32	42.9	66	42	51.5	53	12	19.1
67	34	46.1	90	39	58.1	80	17	35.1
83	32	51.2	102	38	63.7	99	20	41.6
103	33	55.6				123	26	47.7

References

1. Ostwald, W. Studien uber die bildung und umwandlung fester korper. *Z. Phys. Chem.* **22,** 289–330 (1897).
2. Ahn, T. Formation pathways of magnetite nanoparticles by coprecipitation method. *J. Phys. Chem. C* **116,** 6069–6076 (2012).
3. Hansel, C. M., Benner, S. G. & Fendorf, S. Competing Fe(II)-induced mineralization pathways of ferrihydrite. *Environ. Sci. Technol.* **39,** 7147 (2005).
4. Vali, H. et al. Formation of tabular single-domain magnetite induced by *Geobacter metallireducens* GS-15. *Proc. Natl. Acad. Sci. U. S. A.* **101,** 16121–16126 (2004).
5. Thanh, N. T. K., Maclean, N. & Mahiddine, S. Mechanisms of nucleation and growth of nanoparticles in solution. *Chem. Rev.* **114,** 7610–7630 (2014).
